# Home versus Clinic Blood Pressure Monitoring: Evaluating Applicability in Hypertension Management via Telemedicine

**DOI:** 10.3390/diagnostics13162686

**Published:** 2023-08-15

**Authors:** Ali F. Al-Anazi, Rahim Gul, Fahad T. Al-Harbi, Sulaiman A. Al-Radhi, Hamood Al-Harbi, Altigani Altaher, Mohammed M. Al-Harbi, Fahad O. Al-Rashidi, Omer S. Al-Haweeri, Fakhri M. Al-Mutairi, Afyaa A. Al-Riyaee, Fai M. Al-Hotan, Alulu A. Al-Radhi, Hamdan M. Al Shehri, Mohammed S. Alharbi, Naif Saad ALGhasab

**Affiliations:** 1 Department of Adult Cardiology, Prince Sultan Cardiac Center, Buraydah 52366, Qassim, Saudi Arabia; 2 Department of Internal Medicine, Al-Rass General Hospital, Al-Rass 58883, Qassim, Saudi Arabia; 3 College of Medicine, Qassim University, Buraydah 51482, Qassim, Saudi Arabia; 4 Department of Pharmacy, Qassim University Medical City, Buraidah 52571, Qassim, Saudi Arabia; 5 Department of Internal Medicine, Medical College, Najarn University, Najran 55461, Najran, Saudi Arabia; 6 Department of Internal Medicine, Medical College, Ha’il University, Ha’il 55476, Hail, Saudi Arabia

**Keywords:** hypertension, morning blood pressure, telehealth, home monitoring, masked hypertension, ambulatory blood pressure monitoring

## Abstract

Hypertension is a significant public health concern in Saudi Arabia, affecting 28.6% of the population. Despite the availability of effective treatments, optimal blood pressure control is not always achieved, highlighting the need for effective management strategies. This study aimed to evaluate the applicability of home, compared to clinic, blood pressure measurements for managing hypertension in the Qassim region of Saudi Arabia. The study included 85 adults undergoing antihypertensive treatment. Home blood pressure measurements were obtained during the day and the evening using automated oscillometric sphygmomanometers, whereas clinic measurements were taken during clinic hours. Home blood pressure readings were significantly lower than clinic blood pressure readings, with mean differences of 20.4 mmHg and 4.1 mmHg for systolic and diastolic blood pressures, respectively. There was a positive correlation between the clinic systolic and diastolic blood pressures (r = 0.549, *p* < 0.001) and a weak correlation between the daytime home and clinic systolic blood pressures (r = 0.218, *p* < 0.05). This study provides insight into the applicability of home blood pressure monitoring, which may aid in the development of more effective hypertension management strategies, particularly the use of morning home blood pressure monitoring to aid treatment decisions through telehealth medicine.

## 1. Introduction

Hypertension is a significant public health concern, affecting millions of individuals worldwide, including those from the Saudi Arabian population. Approximately 32% and 28.6% of the global and Saudi Arabian populations, respectively, are hypertensive [1,2]. Hypertension significantly increases the risk of stroke, cardiovascular disease, and renal failure, and may, therefore, significantly impact an individual’s quality of life and overall health outcomes [3,4].

Despite the availability of effective treatment options for hypertension, many individuals struggle to achieve optimal blood pressure control. Guideline-directed treatments have been shown to be effective in reducing the incidence of hypertension-related complications, but only 54% of individuals with hypertension achieve their target blood pressure [5,6]. This highlights the need for more effective management strategies and interventions to improve blood pressure control and reduce the risk of hypertension-related complications [7].

The methods available for measuring blood pressure vary in sensitivity and accuracy. They include clinic blood pressure (CBP) measurement, home blood pressure (HBP) measurement, and 24 h ambulatory monitoring [8,9]. It is important to consider conditions, such as the white coat effect and masked hypertension, when measuring blood pressure, and alternative methods may be necessary before adjusting the hypertension therapy [8]. Reliance on clinic blood pressure measurements alone can lead to overtreatment, particularly if the results are overestimated [8,10]. This underscores the need for more comprehensive and accurate blood pressure measurement methods.

Studies exploring the clinical significance of clinic blood pressure measurement accuracy in Saudi Arabia are scarce. However, a study conducted by Mahmud et al. (2020) compared clinic and ambulatory blood pressure monitoring and found that one-third of individuals may have received inappropriate treatment based on the clinic blood pressure measurements [11]. This highlights the need for more comprehensive studies to improve the applicability of blood pressure measurements in clinical and home settings. Additionally, previous studies have indicated that CBP readings are typically higher than HBP readings, with an average difference of 5.3 mmHg for systolic blood pressure (SBP) and 3.1 mmHg for diastolic blood pressure (DBP) [12,13].

Developing effective strategies for managing hypertension in the community requires a thorough understanding of the differences between clinic and HBP measurements. We conducted a comprehensive study in the Qassim region, to identify and examine the variations between these two types of blood pressure measurements among hypertensive individuals. Through this study, we hoped to gain insights into the factors that contribute to the differences between clinic and HBP measurements and to use this information to develop targeted interventions that could improve hypertension management in our community. To summarize, the objective was to improve the health outcomes of individuals with hypertension and to reduce the burden of this condition on the healthcare system.

## 2. Materials and Methods

### 2.1. Study Population

**Figure 1:**
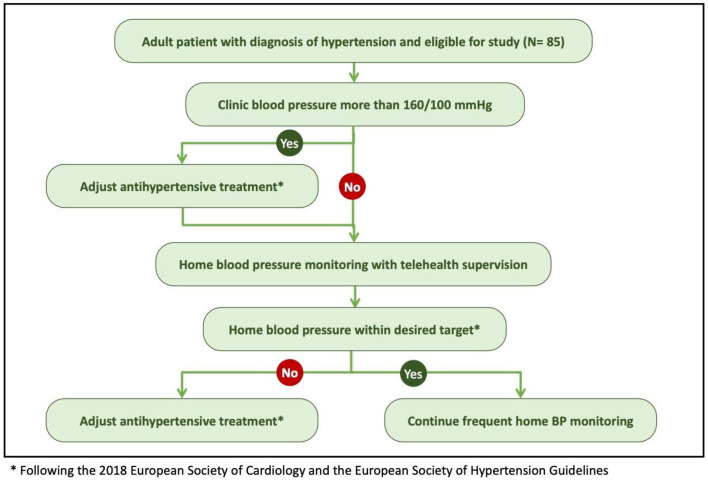
Algorithm for diagnosing and monitoring blood pressure during clinic and home follow-up.

This study included adults diagnosed with hypertension (*n* = 85) and receiving antihypertensive drug treatment at the Prince Sultan Cardiac Center in Qassim, Saudi Arabia (Figure 1). 

### 2.2. Study Design

This was a one-year, prospective, cross-sectional, cohort study conducted from 1 January to 31 December 2020. The study evaluated the applicability of HBP measurements compared to CBP measurements in the management of hypertension.

### 2.3. Inclusion Criteria

The following criteria were used to select participants: (1) age of 18 years or older, (2) newly diagnosed or known hypertension, with hypertension defined as SBP ≥ 140 mmHg and/or DBP ≥ 90 mmHg by the 2018 European Society of Cardiology and the European Society of Hypertension Guidelines, and (3) capable of performing HBP measurements. 

### 2.4. Exclusion Criteria

These included antihypertensive treatment adjustment within three months of enrollment, inconclusive data, or missing data.

### 2.5. Home and Clinic Blood Pressure Measurements

HBP measurements were performed using automated oscillometric sphygmomanometers. The arm circumference was measured, and the appropriate cuff size was selected. Following a 5 min rest period, a blood pressure reading greater than 140/90 mmHg, and at least two readings taken during a clinic visit, patients were instructed on how to use an HBP monitoring device, such as the BEURER BM 85 (EN 60601-1-2) [14,15], Geratherm smart (EN ISO 13485) [16], Omron M6 or Grandway MD5681, to self-measure their blood pressure twice daily (>2 readings), while seated, at home for five consecutive days [17,18,19].

We checked the blood pressure using the same home device at each patient’s primary health clinic to ensure the accuracy of the HBP readings prior to starting the self-measurement.

The first set of measurements were taken within one hour of waking and the second set between 7:00 p.m. and 10:00 p.m. During the follow-up period, patients were contacted after one week to assess their blood pressure control and make any necessary adjustments to the treatments. If the blood pressure was not controlled, further follow-up was scheduled within two weeks for additional management. Once the blood pressure was successfully controlled, follow-up appointments were scheduled at six-month and one-year intervals to ensure blood pressure control in patients using telemedicine.

A data sheet with patient demographics and a schedule for blood pressure measurement results, based on dates and times, were given to the patients, and they were trained to complete it.

### 2.6. Statistical Analyses

Statistical analyses were performed using the SPSS^®^ statistical software package version 18.0 (SPSS Inc., Chicago, IL, USA) for Windows10^®^. The findings of the study were summarized in tables, with the representation of continuous variables as the mean and standard deviation. Categorical variables were reported as numbers and percentages, and the chi-square test was used for comparisons. The comparison in this study focused on male and female patients, considering various factors such as age, body mass index (BMI), nationality, smoking status, newly diagnosed hypertension, and whether medication was adjusted in a home or clinic setting. Matrix correlation was used to determine the associations between clinic SBP/DBP and other clinic/home blood pressure variables. The mean difference between the CBP and HBP results was calculated, and Cohen’s d test was used to determine the effect size. Additionally, further analyses were performed to evaluate the difference between the systolic and diastolic blood pressure measurements conducted in the clinic and at home. Statistical significance was set at a *p*-value of less than 0.05.

## 3. Results

The study included 85 patients, with the majority being older adults (*n* = 59; 69.4%), followed by middle-aged adults (*n* = 24; 28.2%), and young adults (*n* = 2; 2.4%). The patients had a mean age of 64 years (range: 33–91 years). Majority of the patients in the study were classified as obese (*n* = 50; 57.6%), followed by overweight (*n* = 18; 21.2%). The patients had a mean BMI of 26.55 kg/m^2^ (range: 14–39 kg/m^2^). A significant *p*-value was observed for BMI and sex across the study population (*p* = 0.0098). The male-to-female ratio was approximately equal, with 44 (52%) male patients and 41 (48%) female patients (see Table 1 and Table 2 for further details).

Among the 85 patients who participated in the study, 35 (41%) had their antihypertensive medication adjusted following the CBP reading, while 41 (48%) had their medication adjusted after HBP readings. As indicated in Table 1, 81 patients (95%) had a prior diagnosis of hypertension, while 4 (5%) were newly diagnosed. Most of the patients (77, 90.5%) were non-smokers, while there were five (6%) ex-smokers, and three (3.5%) current smokers.

Forty-seven patients had a mean BMI of 26.5, with a mean weight of 71.30 kg and a mean height of 162.55 cm. The mean clinic systolic blood pressure (CSBP) was 159.45 mmHg, while the mean clinic diastolic blood pressure (CDBP) was 79.14 mmHg. The mean morning home systolic blood pressure (MHSBP) and morning home diastolic blood pressure (MHDBP) were 140.69 mmHg and 75.76 mmHg, respectively. The mean evening home systolic blood pressure (EHSBP) and evening home diastolic blood pressure (EHDBP) were 138.95 mmHg and 75.20 mmHg, respectively. The mean home systolic (averaged across both daytime and evening readings) and diastolic blood pressures were 138.99 mmHg and 75.03 mmHg, respectively. The mean difference between the clinic and home SBPs was 20.46 mmHg, while the mean difference between the clinic and home DBPs was 4.11 mmHg (Table 2).

Table 3 shows the correlation matrix among various blood pressure measurements, including CSBP, CDBP, home SBP during the day, home DBP during the day, home SBP in the evening, home DBP in the evening, home day–evening SBP mean, and home day–evening DBP mean.

We found a significant positive correlation (r = 0.549, *p* < 0.001) between the CSBP and CDBP, indicating that an increase in CSBP is associated with an increase in CDBP. Additionally, there was a weak positive correlation (r = 0.218, *p* < 0.05) between the home systolic blood pressure (HSBP) during the day and the CSBP, suggesting that a higher HSBP during the day might be associated with a higher CSBP.

Furthermore, we found a significant positive correlation (r = 0.786, *p* < 0.001) between the home diastolic blood pressure (HDBP) during the day and that during the evening, indicating that a higher HDBP during the day may be associated with a higher HDBP during the evening. As shown in Table 3, the study found a strong positive correlation between the home mean (day–evening) SBP and home SBP during both the day and evening (r = 0.941, *p* < 0.001; r = 0.885, *p* < 0.001, respectively), as well as between the mean day–evening DBP and home DBP during the day and evening (r = 0.948, *p* < 0.001; r = 0.842, *p* < 0.001, respectively).

For SBP, the mean difference between the clinic and home results was 20.4 mmHg (CSBP: M = 159.4, SD = 16.09; HSBP: M = 139.0). This difference was statistically significant (t (84) = 8.43, *p* < 0.001), indicating that HSBP was significantly lower than CSBP. The effect size, measured using Cohen’s d, was large (d = 0.92), indicating a substantial difference between the two results.

For DBP, the mean difference between the clinic and home results was 4.1 mmHg (CDBP: M = 79.1, SD = 11.80; HDBP: M = 75.0). This difference was also statistically significant (t (84) = 2.91, *p* = 0.005), indicating that HDBP was significantly lower than CDBP. The effect size was smaller (d = 0.32), indicating a less substantial difference between the two results compared to the SBP (Table 4).

Table 5 shows the distribution of SBP and DBP results in the clinic and home settings. A larger proportion of patients had higher SBP readings, of 160 mmHg or above, in the clinic (*n* = 71; 83.6%), while in the home setting, the majority (*n* = 57; 67.1%) had SBP readings of 145 mmHg or above. The data also indicate that most patients in the clinic had a DBP of less than 80 mmHg (*n* = 55; 64.7%). However, a higher proportion of patients in the home setting had DBP readings of less than 80 mmHg (*n* = 64; 75.3%).

## 4. Discussion

Our study findings demonstrated a significant discrepancy between blood pressure readings obtained in clinic and home settings, particularly regarding SBP. While the results for both SBP and DBP were not consistent across both settings, the analysis revealed a noteworthy difference in SBP readings. These results underscore the importance of considering the context in which blood pressure is measured when interpreting blood pressure values, as the measurement methodology can have a substantial effect on the results. These findings suggest that clinic and home monitoring measurements may be independent of each other, and that home monitoring may provide a more accurate representation of a patient’s blood pressure over time. Our study holds great importance for the management of patients, particularly those with elevated HBP values.

Home monitoring of morning blood pressure is considered superior to ambulatory or clinic measurements, especially when taken over a period of several days, owing to its greater reproducibility and stronger correlation with actual blood pressure levels, as opposed to evening measurements [20]. The findings shown in Table 3 and Table 4 suggest that blood pressure monitoring is independent, and substantially varied between clinic and home settings in the study cohort (*n* = 85, *p* < 0.001), which has significant implications for the management of hypertension. These results indicate that clinic measurement alone may not provide an accurate representation of a patient’s blood pressure and that home monitoring may be a more reliable and precise method of monitoring blood pressure over time. Therefore, incorporating home monitoring via telemedicine into the management and treatment of patients with hypertension may be a more effective approach for achieving optimal blood pressure control and reducing the risk of associated complications (Table 4).

### 4.1. Difference between CSBP and HSBP

In our study, the mean difference in SBP readings was 20.46 mmHg, which is higher than the international norm. A systematic review and meta-analysis [21] also identified disparities between clinic and home SBP values, with mean clinic SBP values being significantly higher than MHSBP values, by 3.79 mmHg (95% CI, 2.77–4.80). The differences were more pronounced in Europe (6.53 mmHg (95% CI, 4.10–8.97)) than in Asia (2.70 mmHg (95% CI, 1.74–3.66)), with the region being a significant predictor of differences [22].

The divergence in blood pressure readings between clinic and home settings in our study could be attributed to several factors. One potential factor is the difference in our study population compared to previous studies. Additionally, our study revealed a higher proportion of elevated blood pressure readings, which could also contribute to the observed divergence. The “white coat effect”, where the anxiety of being in a medical environment can lead to a temporary increase in blood pressure, is one reason. Additionally, blood pressure can fluctuate throughout the day and be influenced by various factors, such as physical activity, diet, and stress levels. Home blood pressure readings, taken in a more relaxed environment and at different times of the day, may provide a more accurate representation of a person’s overall blood pressure levels [17,23]. These findings underscore the significance of HBP readings as a reliable assessment tool for identifying such differences.

The higher mean difference observed in our study could be attributed to the larger proportion of patients in our CBP sample with high SBP readings of 160 mmHg or more (*n* = 71, 83.6%), as well as for DBP readings, as shown in Table 5. The 2017 ACC guidelines suggest that a mean difference of 15 mmHg for SBP and 5 mmHg for DBP can be anticipated between home and clinic readings in patients with SBP greater than 160 mmHg [24]. These findings highlight the importance of considering patients’ blood pressure levels when interpreting differences between clinic and home readings and the need for regular monitoring of blood pressure in patients with hypertension to achieve optimal control and reduce the risk of associated complications.

### 4.2. Telehealth in the Management of Hypertension

We observed that 89% of the patients (*n* = 76) received blood pressure management either in the clinic or at home after obtaining BP readings, while approximately half of the study population (*n* = 41; 48%) had their medication adjusted through landline, mobile telephone/modem transmission, or the internet (Table 1). Telehealth has emerged as a vital and effective tool for managing hypertension, facilitating remote monitoring of vital signs such as blood pressure, medication adherence, and for the provision of education on lifestyle and risk factors. This approach can enhance access to care and empower patients to take responsibility for their health [25].

HBP monitoring equipment has been shown to be helpful for the management of hypertension, despite its heterogeneity, but strong and consistent evidence is presented [26,27,28]. Although home telemonitoring of blood pressure can be helpful, it may not provide a comprehensive picture of an individual’s blood pressure patterns, unlike ambulatory monitoring, which tracks blood pressure throughout the day and evening and provides information on circadian rhythms. 

One study suggested that the most effective healthcare model for telehealth in hypertension management should incorporate remote monitoring and the transmission of vital signs, particularly blood pressure, medication adherence, and education on lifestyle and risk factors, with video consultation as an optional feature [25]. Telehealth has also proven to be a valuable tool during the COVID-19 pandemic, allowing for the management of patients that were in isolation due to lockdown or shielding [25,29]. Overall, telehealth has demonstrated significant potential for enhancing the management of hypertension and other chronic conditions, as evidenced by our follow-up in 2020.

A recent meta-analysis provided significant evidence of the therapeutic benefits of interventions, particularly regarding self-monitoring of blood pressure. The analysis found that when self-monitoring was combined with co-interventions, such as education or lifestyle counseling, it resulted in greater reductions in SBP/DBP compared to usual care controls, with reductions of up to 6.1 mmHg [30]. Additionally, self-monitoring was found to lower blood pressure regardless of the number of hypertension-related co-morbidities. However, in cases with conditions such as obesity or stroke, self-monitoring may only be effective when combined with high-intensity co-interventions [31]. In our study population, frequent communication with patients through voice/video calls or smartphone apps significantly increased patient self-awareness, which may have helped in controlling blood pressure during later follow-up periods.

Another systematic review reported that telehealth interventions led by nurses resulted in a significant decrease in blood pressure levels, especially SBP, in the intervention groups. The review also revealed positive effects on reducing cholesterol levels, increasing the consumption of fruits and vegetables, promoting physical activity, and enhancing adherence to medication. Furthermore, the nurse-led interventions were found to have a positive impact on hypertension awareness, self-efficacy, and self-control [32]. Using a digital intervention in poorly controlled hypertension showed that HBP digital interventions achieved targets with no increase in adverse effects, suggesting that digital interventions reduce clinical inertia, lead to optimization of treatment, have lower marginal costs, and enable patients to play a more active role in controlling their blood pressure [33,34].

## 5. Conclusions

In summary, our study highlights the significance of telehealth and systolic HBP monitoring in the management of hypertension. We found that the clinic blood pressure readings were consistently higher than the home blood pressure readings. Physician- or nurse-led interventions, including the promotion of medication adherence and education on lifestyle factors, are effective in controlling and managing hypertension. Patients should be educated on the importance of HBP monitoring, which is equally significant in hypertension management. Telehealth interventions can enhance access to care and empower patients to take control of their health, making them valuable tools for hypertension management.

## Figures and Tables

**Table 1:** Baseline characteristics of the study population.

Variable	Sub-Category	Overall (*n* = 85)	Male (*n* = 44)	Female (*n* = 41)	χ^2^	*p*-Value
Age (years)	Young age (<40)	2 (2.4%)	2 (2.4%)	0 (0%)	0.6064	0.7384
	Middle-aged adult (40–60)	24 (28.2%)	11 (13%)	13 (15.2%)		
	Old adult (>60)	59 (69.4%)	31 (36.4%)	28 (33%)		
BMI (kg/m^2^)	Underweight (<18)	2 (2.4%)	1 (1.2%)	1 (1.2%)	11.3883	0.0098
	Normal (18–24)	15 (18.8%)	9 (11.7%)	6 (7.1%)		
	Overweight (>24)	18 (21.2%)	15 (17.7%)	3 (3.5%)		
	Obese (>29)	50 (57.6%)	19 (21.2%)	31 (36.4%)		
Nationality	Saudi	84 (98.8%)	43 (50.6%)	41 (48.2%)	0.0011	0.9734
	Egyptian	1 (1.2%)	1 (1.2%)	0 (0%)		
Smoking History	Yes	3 (3.5%)	3 (3.5%)	0 (0%)	3.9804	0.1366
	No	77 (90.5%)	36 (42.3%)	41 (48.2%)		
	Ex-smoker	5 (6%)	5 (6%)	0 (0%)		
Newly diagnosed hypertension		4 (4.7%)	1 (1.2%)	3 (3.5%)	1.2043	0.2724
Medication adjusted in clinic	CBP Reading	35 (41%)	16 (21%)	17 (20%)	0.2324	0.6297
Medication adjusted at home	HBP Reading	41 (48.2%)	21 (24.7%)	20 (23.5%)	0.0094	0.9226

**Table 2:** Baseline mean clinical characteristics of the study population.

Variable	*N*	Mean	Median	SD	Range	Minimum	Maximum	Skewness	Kurtosis
Age (years)	81	64.43	64.00	11.85	58.00	33.00	91.00	−0.20	−0.11
BMI (kg/m^2^)	47	26.55	27.00	4.77	25.00	14.00	39.00	0.08	0.97
Weight (kg)	47	71.30	70.00	12.65	67.00	33.00	100.00	−0.05	1.27
Height (cm)	47	162.55	162.00	7.01	29.00	148.00	177.00	−0.05	−0.55
Duration HBP Monitoring	85	4.87	5.00	0.75	6.00	1.00	7.00	−2.53	11.18
Clinic SBP	85	159.45	160.00	16.09	75.00	125.00	200.00	0.06	−0.12
Clinic DBP	85	79.14	80.00	11.80	53.00	57.00	110.00	0.33	−0.53
Home SBP (Day)	84	140.69	141.13	16.83	91.00	92.00	183.00	−0.11	0.88
Home DBP (Day)	84	75.76	75.63	9.77	58.00	54.00	112.00	0.54	1.18
Home SBP (Evening)	85	138.95	140.00	15.92	73.70	97.00	170.70	−0.37	−0.36
Home DBP (Evening)	85	75.20	74.00	9.27	46.50	57.50	104.00	0.63	0.54
Home Day–Evening SBP (mean)	85	138.99	141.50	17.75	118.00	54.50	172.50	−1.35	5.34
Home Day–Evening DBP (mean)	85	75.03	74.50	9.75	68.00	40.00	108.00	0.08	2.15
Clinic–Home SBP (difference)	85	20.46	17.00	22.36	124.50	−19.00	105.50	0.86	1.54
Clinic–Home DBP (difference)	85	4.11	6.00	13.02	88.00	−38.00	50.00	0.10	1.79

Note: BMI = body mass index, SD = standard deviation, SBP = systolic blood pressure, DBP = diastolic blood pressure.

**Table 3:** Correlation matrix of various blood pressure measurements.

Variable	1	2	3	4	5	6	7	8
Clinic SBP	—							
Clinic DBP	0.549 ***	—						
Home SBP (Day)	0.218 *	0.126	—					
Home DBP (Day)	0.177	0.331 **	0.349 **	—				
Home SBP (Evening)	0.062	0.065	0.754 ***	0.199	—			
Home DBP (Evening)	0.059	0.338 **	0.150	0.786 ***	0.240 *	—		
Home Day–Evening SBP (mean)	0.129	0.045	0.941 ***	0.296 **	0.885 ***	0.154	—	
Home Day–Evening DBP (mean)	0.115	0.282 **	0.267 *	0.948 ***	0.298 **	0.842 ***	0.419 ***	—

Note: SBP = systolic blood pressure; DBP = diastolic blood pressure; * *p* < 0.05, ** *p* < 0.01, *** *p* < 0.001. The strength of positive correlations is indicated by different colors and a varying number of asterisks.

**Table 4:** Mean difference between clinic and home blood pressures.

Mean Difference between Clinic and Home Blood Pressures						
Variable	*n*	M	SD	T (84)	*p*	Cohen’s d
CSBP	85	159.4	16.09	8.43	<0.001	0.92
HSBP	85	139.0	17.75			
CDBP	85	79.1	11.80	2.91	0.005	0.32
HDBP	85	75.0	9.75			

CSBP: clinic systolic blood pressure; HSBP: home systolic blood pressure; CDBP: clinic diastolic blood pressure; HDBP: home diastolic blood pressure.

**Table 5:** Frequency and percentage of systolic and diastolic blood pressure measurements conducted in the clinic and at home.

Clinic Blood Pressure (CBP)						Home Blood Pressure (HBP)					
Systolic BP			Diastolic BP			Systolic BP			Diastolic BP		
mmHg	*n*	%	mmHg	*n*	%	mmHg	*n*	%	mmHg	*n*	%
<120	0	0.0	<80	55	64.7	<120	8	9.4	<80	64	75.3
120	0	0.0	80	21	24.7	120	1	1.2	80	9	10.6
130	4	4.7	80			130	13	15.3	80		
140	10	11.8	90	7	8.2	135	6	7.1	85	8	9.4
160	40	47.1	100	2	2.4	145	27	31.8	90	4	4.7
>160	31	36.5	>100	0	0	>145	30	35.3	>90	0	0

CBP: clinic blood pressure; HBP: home blood pressure; BP: blood pressure.

## Data Availability

Data are available upon request from the authors.

## Abbreviations

CBP: clinic blood pressure; HBP: home blood pressure; SBP: systolic blood pressure; DBP: diastolic blood pressure; BMI: body mass index; CSBP: clinic systolic blood pressure; CDBP: clinic diastolic blood pressure; MHSBP: morning home systolic blood pressure; MHDBP: morning home diastolic blood pressure; NHSBP: evening home systolic blood pressure; NHDBP: evening home diastolic blood pressure; HSBP: home systolic blood pressure.
